# An Arabidopsis Prolyl 4 Hydroxylase Is Involved in the Low Oxygen Response

**DOI:** 10.3389/fpls.2021.637352

**Published:** 2021-03-15

**Authors:** Anna Konkina, Mariola Klepadlo, Abdellah Lakehal, Zein El Zein, Afroditi Krokida, Mina Botros, Michail Iakovidis, Pavel Chernobavskiy, Mohamed Elfatih Zerroumda, George Tsanakas, Nikos Petrakis, Athanasia-Maria Dourou, Panagiotis Kalaitzis

**Affiliations:** Department of Horticultural Genetics and Biotechnology, Mediterranean Agronomic Institute of Chania, Chania, Greece

**Keywords:** *Arabidopsis thaliana*, prolyl-4-hydroxylases, anoxia, hypoxia, AGPs, FLAs, ADH, pDc

## Abstract

Plant responses to flooding, submergence and waterlogging are important for adaptation to climate change environments. Therefore, the characterization of the molecular mechanisms activated under hypoxic and anoxic conditions might lead to low oxygen resilient crops. Although in mammalian systems prolyl 4 hydroxylases (P4Hs) are involved in the oxygen sensing pathway, their role in plants under low oxygen has not been extensively investigated. In this report, an Arabidopsis *AtP4H3* T-DNA knock out mutant line showed higher sensitivity to anoxic treatment possibly due to lower induction of the fermentation pathway genes, *ADH* and *PDC1*, and of sucrose synthases, *SUS1* and *SUS4*. This sensitivity to anoxia was accompanied by lower protein levels of AGPs-bound epitopes such as LM14 in the mutant line and induction of extensins-bound epitopes, while the expression levels of the majority of the AGPs genes were stable throughout a low oxygen time course. The lower AGPs content might be related to altered frequency of proline hydroxylation occurrence in the *p4h3* line. These results indicate active involvement of proline hydroxylation, a post-translational modification, to low oxygen response in Arabidopsis.

## Introduction

Oxygen is important for cellular respiration in all eukaryotes, including plants, which often encounter oxygen deficiency conditions during their life cycle such as root waterlogging and submergence and as a result have evolved various mechanisms of sensing and response to oxygen availability (Gibbs and Greenway, 2003; Loreti and Perata, 2020). Oxygen deprivation triggers various changes at different levels, such as metabolic shifts (Roberts et al., 1984; Drew, 1997; Planchet et al., 2017), alterations in gene expression (Branco-Price et al., 2005; Loreti et al., 2005; Vlad et al., 2007b), post-translational modifications (Iacopino and Licausi, 2020), and morphological changes (Perata and Alpi, 1993; Eysholdt-Derzsó and Sauter, 2017).

The activation of glycolysis for ATP production (Perata and Alpi, 1993; Gibbs and Greenway, 2003) and fermentative metabolism due to the upregulation of pyruvate decarboxylase (PDC) and alcohol dehydrogenase (ADH), leads to increased plant tolerance (Knox et al., 1995; Ismond et al., 2003; Kürsteiner et al., 2003). Moreover, knock-out mutants of Arabidopsis *PDC1* and *PDC2* resulted in higher susceptibility to submergence (Mithran et al., 2014), and mutants of rice and Arabidopsis *ADH* suppressed seed germination under anoxia (Jacobs et al., 1988; Matsumura et al., 1995; Banti et al., 2008). The glycolytic flow is maintained by the induction of energy efficient sucrose synthase (SUSs) genes such as *SUS1* and *SUS4* in Arabidopsis which were shown to be up-regulated under hypoxia (Santaniello et al., 2014).

In plants, oxygen sensing is mediated by the regulation of protein stability of group VII ERF (Ethylene Response Factor) cluster of transcription factors via the N-end rule pathway (Gibbs et al., 2011; Weits et al., 2014). A major component of this pathway is the Plant Cysteine Oxidase genes (PCO) which oxidize a Cys at the N-terminus of the VII ERFs polypeptide in an oxygen dependent manner, thus targeting VII ERFs to proteasomal degradation (Weits et al., 2014). The VII ERFs control the expression of anaerobic genes by binding to Hypoxia Response Promoter Element motifs present in anaerobic genes such as *ADH*, *PDC*, *LBD41* (LOB Domain-Containing Protein 41), *HRE1* as well as *HRE2* (Hypoxia Responsive ERF 1 and 2), *HRA1* (Hypoxia Response Attenuator) (Gasch et al., 2016; Giuntoli and Perata, 2018).

In the metazoans, the prolyl-4-hydroxylases (PHD, prolyl-4-hydroxylase domain) act as oxygen sensors by hydroxylation of HIF1α (Hypoxia Inducible Factor 1A) transcription factor (Wang and Semenza, 1993; Kaelin and Ratcliffe, 2008). Under normoxia PHDs mediate hydroxylation of proline in HIF1α, thus targeting it to proteasomal degradation (Pugh and Ratcliffe, 2003). Under hypoxia, HIFα accumulates, dimerizes with an HIFβ family member, translocates to the nucleus, and transcriptionally activates more than 100 genes (Mcneill et al., 2002; Kaelin and Ratcliffe, 2008; Myllyharju and Koivunen, 2013).

Plant prolyl-4-hydroxylases (P4H) are non-heme proteins belonging to the 2-oxoglutarate-dependent dioxygenase family (2-ODD) requiring molecular oxygen to catalyze proline hydroxylation of Hydroxyproline Rich Glycoproteins (HRGPs) and additional proteins (Clifton et al., 2006; Farrow and Facchini, 2014). This important post-translational modification (PTM) was detected in extensins (Velasquez et al., 2011, 2012), arabinogalactan proteins (AGP) (Schultz et al., 2004), as well as secreted peptide hormones (Kondo et al., 2006; Stührwohldt et al., 2020) and tyrosine-sulfated glycopeptides (Amano et al., 2007). In tomato (*Solanum lycopersicum*) only the *SlAGP4* transcript abundance increased in response to an anoxic time course in mature green fruit pericarp tissue, while AGPs-bound epitopes were either constitutively expressed or upregulated (Fragkostefanakis et al., 2012). In addition to their involvement in various abiotic stresses in plants (Lamport et al., 2006; Mareri et al., 2019), fasciclin-like arabinogalactans (FLAs) and extensins (EXT) showed alterations in transcript levels in a deep-water rice cultivar of high tolerance to anaerobiosis, while three FLAs and two EXTs were upregulated (Minami et al., 2018). Several Arabidopsis AGPs were differentially expressed in a tissue-specific manner in roots under hypoxia and anoxia (Mustroph et al., 2009), while 22 Arabidopsis HRGPs, including five AGPs and five extensins, were identified *in silico* to exhibit alterations in expression patterns under anoxia (Schultz et al., 2002; Vlad et al., 2007a). Interestingly, five out of 13 Arabidopsis P4Hs were co-expressed with various AGPs suggesting putative interaction (Showalter et al., 2010). These data suggest possible involvement of AGPs in hypoxic and anoxic adaptation. Moreover, overexpression of Arabidopsis AtP4H1 resulted in higher expression of hypoxia-induced genes such as ADH1, PDC1, PDC2, and SUS1 (Asif et al., 2009).

In this study, the physiological role of AtP4H3 under hypoxic and anoxic conditions was investigated. An *AtP4H3* T-DNA knock out mutant line exhibited reduced survival percentages under anoxia probably due to lower induction of the fermentation pathway genes, while the AGPs transcript abundance and bound-epitopes remained stable under hypoxic conditions. Moreover, analysis of Arabidopsis lines transformed with a construct of *AtP4H3* promoter directing GUS expression indicated tissue specific expression mainly in root stele and columella cells, leaf tips and stipules under hypoxia.

## Materials and Methods

### Plant Material and Growth Conditions

The *A. thaliana* knock-out mutant line of *AtP4H3* (*At1g20270*) N576446 (SALK_076446) was obtained from the European Arabidopsis Stock Centre (NASC)^1^. Seeds of *Arabidopsis thaliana* p4h3-2 mutants were surface-sterilized (30 s 100% ethanol followed by 5 min in 50% bleach), rinsed and allowed to imbibe at 4°C. After 4 days, seeds were transferred to plates with solid MS media (Appendix), and placed in a growth chamber (Snijders Scientific) in vertical orientation under long-day conditions [16 h light (100% intensity) and 8 h dark] with 70% humidity at 22°C during the light cycle and 16°C during the dark cycle. After 10 days, surviving seedlings were transferred to Murashige and Skoog (MS) medium without antibiotic selection. Two weeks later, plants were transferred into jars, and then to soil. Each plant was individually labeled, bagged, and placed separately to avoid cross-pollination.

### DNA Extraction

To select for mutant plants following kanamycin selection on MS plates, 200 mg of leaf tissue from 6-week-old plants was frozen with liquid nitrogen and ground by pestle and mortar. 500 μl of DNA extraction buffer (200 mM Tris–HCl pH = 8.0; 250 mM NaCl; 25 mM EDTA pH = 8.0; 0.5% SDS) was added and samples were mixed by inversion several times. Then, an equal volume of phenol: chloroform 1:1 was added to the samples, vortexed for 20 s, centrifuged at 13000 rpm for 10 min and supernatants were transferred to new tubes. Cold isopropanol in 1:1 volume was added, samples were mixed by inversion and placed in −20°C for 1 h. Samples were centrifuged, washed with 70% ethanol, air-dried and resuspended in 150 μl of sterile distilled H_2_O. 20 μl of RNase A (10 mg/ml) was added and samples were then incubated for 1 h at 37°C. An equal volume of chloroform was added again, vortexed and centrifuged for 10 min. The supernatants were transferred to new tubes and washed with 2.5:1 volumes of 100% and 70% ethanol. Dried pellets were resuspended in 50 μl of sterile distilled H_2_O and stored at −20°C. DNA was quantified by an IMPLEN NanoPhotometer^TM^ Pearl.

### RNA Extraction and cDNA Synthesis

Total RNA was isolated from roots of 7 day-old Arabidopsis Col-0 plants using PureLink^®^ RNA Mini Kit by Ambion. 200–300 mg of frozen tissue was homogenized in liquid nitrogen and following steps were done according to the PureLink^®^ RNA Mini Kit instructions. Quality control for RNA was performed with GelRed^®^ Nucleic Acid Gel Stain (Biotium) on a 1.5% denaturing agarose gel electrophoresis with 2 μg RNA and a NanoPhotometer (NanoPhotometer^TM^ Pearl, Implen). cDNA was synthesized using 5 μg of RNA with oligo (dT)_15_ primers and the SuperScript^®^ Synthesis VILO cDNA kit (Invitrogen) according to the manufacturer’s guidelines.

### Western Blotting

A modified protocol based on Woodson and Handa (1987) was used to extract total protein from wild type and *p4h3* plants (Woodson and Handa, 1987). Plant tissue was ground in liquid nitrogen and lysed afterward (Woodson and Handa, 1987). Extracted proteins were separated by sodium dodecyl sulfate-polyacrylamide 12% (pH 8.8) gel electrophoresis (SDS-PAGE) (Sambrook and Russell, 2006). The gel was transferred onto polyvinyl fluoride membrane (MILLIPORE Immobilon-P, 0.45 mm pore size, IPVH00010) at a constant current of 35 V for 2 h. Blots were soaked in blocking solution containing 5% non-fat dry milk (NFDM) in Tris Buffered Saline (TBS) solution [10 mM Tris HCl, 150 mM NaCl, 0.05% (v/v) Tween-20, 0.01% (w/v) Na Azide, pH 8.0] with shaking at 4°C for 3 h. Then membranes were incubated with LM14 (Moller et al., 2008), JIM20 (Knox et al., 1995), or MAC207 (Pennell et al., 1989) (Paul Knox Cell Wall Lab, University of Leeds, United Kingdom) rat monoclonal antibodies in 2.5% NFDM containing TBS-Tween solution at 4°C overnight. The Actin (Agrisera AB, Vännäs, Sweden) was used as a loading control. The bound antibodies were labeled with the Goat anti-Rat IgG Horse radish peroxidase (HRP conjugate) (MILLIPORE), washed and incubated with a chemiluminescent solution (SuperSignal West Pico Chemiluminescent Substrate, Thermo Scientific, 34077). After 1 min incubation, the membranes were exposed to X-Ray film.

### Generation of Transgenic Plants and *Agrobacterium*-Mediated Transformation

Primers for sequencing and genotyping mutant lines were designed based on using http://signal.salk.edu/tdnaprimers.2.html and http://primer3.ut.ee/, respectively (Supplementary Table 1). To generate *35S:P4H3* transgenic plants, we delimited the open reading frame of *AtP4H3* using the ORF Finder^2^. The DNA fragment was subcloned using the Gateway recombination technology (TOPO Cloning© system) into pCR©II-TOPO© vector according to the manufacturer’s instructions (INVITROGEN)^3^. Plasmids were verified by digestion and transferred to the pK7WG2D Gateway-compatible destination binary vector^4^ by recombination reactions. The generation of *P4H3pro:GFP:GUS* transgenic lines comprised of the amplification of a 1991 bp *AtP4H3* promoter region upstream of the translation initiation codon of *AtP4H3* by using Col-0 genomic DNA as template. PCR products were subcloned using the Gateway recombination technology (TOPO Cloning© system) into pCR©II-TOPO© vector according to the manufacturer’s instructions (INVITROGEN, see text footnote 3). Plasmids were verified by digestion and transferred to the pKGWFS7.0 Gateway-compatible binary vector (see text footnote 4) by recombination reactions. The *AtP4H3* gene construct comprising the entire genomic sequence of 3497 bp for generating complementation lines was amplified from genomic DNA (wild type Col-0) and subcloned into pCR©II-TOPO© vector according to the manufacturer’s instructions (INVITROGEN, see text footnote 3) using the Gateway recombination technology (TOPO Cloning© system). Plasmids were verified by digestion and transferred to the pBGWFS7.0 Gateway-compatible binary vector (see text footnote 4) by recombination reactions. *Agrobacterium tumefaciens* strain GV3101 (pMP90) was used for transforming Arabidopsis plants (Zhang et al., 2006). Independent transformants on kanamycin selection were confirmed by PCR (Supplementary Table 1).

### GUS Visualization for *AtP4H3* Localization

Localization of *AtP4H3* promoter activity was monitored in 8-day old seedlings growing on MS medium containing *P4H3pro:GUS:GFP*. The staining procedure was performed according to Li (2011). For histochemical detection of GUS activity, 7-day old seedlings were collected in Eppendorf tubes and placed on ice along with cold 90% acetone. Following 20 min incubation at 37°C, acetone was removed and plants were washed with rinsing buffer. Then, a staining buffer with X-Gluc was added to each tube and seedlings were placed under vacuum for 5 min. Then the samples were infiltrated under vacuum, on ice, for 30 min and incubated at 37°C under darkness overnight. After an overnight incubation staining buffer was removed and seedlings were washed for 30 min consecutively in 20%, 35%, and 50% ethanol. FAA fixative was then added and seedlings were incubated for another 30 min. FAA was removed, 70% ethanol was added and seedlings were stored at 4°C. Images were collected under an optical microscope Leica MZ FL III at 10x objective with a DC 300F digital camera.

### Hypoxia and Anoxia Treatment

Hypoxia and anoxia conditions were replicated as outlined before (Vlad et al., 2007a). Seven day old seedlings were treated through an open system in Petri dishes with 1.5% O_2_ plus N_2_ (hypoxia) or 100% N_2_ (anoxia) in the dark at room temperature. The Petri dishes were placed in 1 L airtight jars connected in parallel and a 40–45 ml per min gas stream was passed through the system. Concentrations for O_2_ and CO_2_ in the outlet stream were monitored using a CO_2_/O_2_ analyzer (PCO_2_; Gas Data Ltd., Coventry, United Kingdom). Air-treated control plants were left under aerobic conditions. Time-course measurements began when the jars were fully purged with gas. For each time point (0-untreated/control, 1, 2, 4, and 6 h), samples were taken for roots and shoots separately, flash frozen in liquid nitrogen and stored at −80°C.

### Anoxia Tolerance Assay

An anoxia survival assay was performed as outlined previously (Vlad et al., 2007a). Agar plates containing 7 day old seedlings were transferred in plates with anoxia (100% N_2_) for 7.5 h in the dark. Plates were then transferred to a growth chamber for post-anoxic recovery (16 h/8 h, 22°C/16°C, and light/dark photoperiod). Survival rates were measured at 3–4 days post-treatment and averages were taken from three biological replicates. Each replicate comprised of three technical replicates, with each replicate consisting of at least 50 plants for each line. Data were subjected to statistical analysis based on the regression analysis generalized linear model, using GenStat 12.1 software.

### Real Time (qPCR) and Reverse Transcription PCR (RT-PCR)

Real time PCR for Arabidopsis gene expression in response to hypoxia 1.5% O_2_ was conducted on a CFX Connect machine (BIORAD^®^) using SYBR^®^ Select Master Mix (Applied Biosystems^®^). Specificity of primers was determined by dissociation kinetics for PCR products by the end of each run and subsequent analysis of these products using 2% agarose gel electrophoresis stained with GelRed (Biotium). Standard curves were established for each gene using serially diluted cDNA (8, 4, 2, and 1 μg). A linear relationship between the threshold cycle and the log of the starting cDNA concentration was determined for each gene in order to check the efficiency of the PCR. The Arabidopsis Actin gene (*ACTIN2* – *At3g18780*) was used as an internal control.

### Statistical Analysis

For progeny test of seed stocks, one hundred seedlings were tested for kanamycin resistance to observe deviations from Mendelian segregation ratios using Chi-square (χ^2^) statistical tests. Gene expression data were analyzed using the 2^–ΔΔCt^ method (Livak and Schmittgen, 2001) and presented as relative levels of gene expression. To determine relative fold differences for each sample, the Ct value for each gene was normalized to the Ct value for *ACTIN2*.

All measurements were conducted in *triplicate* (three technical replicates per biological replicate and three biological replicates) and results are expressed as mean ± standard errors of the relative expression (SEs). Then, all the data from the pairwise ΔCt of each technical replicate, were subjected to statistical analysis of variance (ANOVA) and a *post hoc* multicomparison test (Tukey’s honest significant difference criterion, 95% confidence interval) by using the statistics toolbox of MATLAB (The MathWorks Inc., Natick, MA, United States). The comparisons were contacted to identify the statistically significant differences between wild type (WT) and the p4h3-2 line, at each timestep of the time-course experiment, respectively. Differences at *P* < 0.05 were considered to be significant.

The reason why in some cases the expression between the control and the mutant line is not statistically significant, even though the expression levels with the error bars do not overlap, is that the statistical analysis was performed on the pairwise ΔCt values for each technical replicate of all three biological replicates. This methodology was selected in order to include all the data and not only the mean values of the three biological replicates (Goni et al., 2009; Pabingera et al., 2014).

## Results

### *AtP4H3* Contributes Toward Oxygen Deprivation Tolerance in Arabidopsis

The Arabidopsis genome comprises 13 putative P4Hs with various patterns of expression under anoxia, hypoxia and mechanical wounding (Vlad et al., 2007a). Only *AtP4H*3 was induced under anoxia and two hypoxic concentrations of 1.5% and 5% O_2_, while *AtP4H4* was expressed under both hypoxic conditions (Vlad et al., 2007a).

**FIGURE 1:**
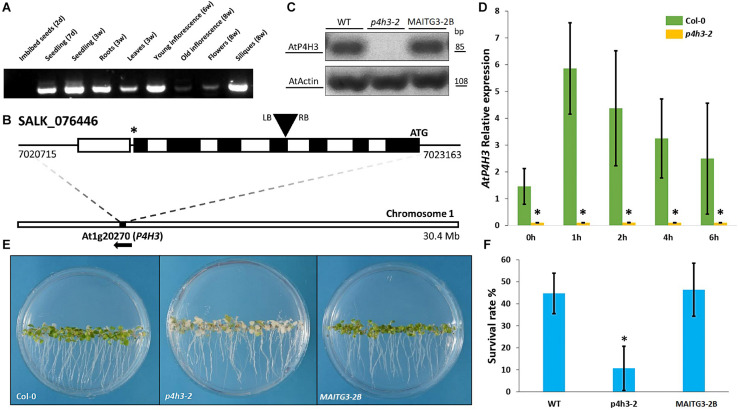
Effects of *AtP4H3* on oxygen deprivation tolerance. **(A)** Tissues-specific expression of *AtP4H3* gene in *Arabidopsis thaliana* (Col-0) by using reverse transcription-PCR. **(B)** T-DNA mutant line insertion schematic of the genomic region corresponding to P4H3. The positions of the T-DNA insertion (black triangle), the initiating codon (ATG), and the stop codon (*) are indicated, along with the location and direction of the gene (*At1g20270*) on chromosome 1. Genomic *AtP4H3* sequences are represented by exons (black boxes), introns (white boxes), and untranslated regions (black line). The T-DNA orientation is indicated by left (LB) and right (RB) borders. Coordinates are in bp and Mb at the gene and chromosome level, respectively. Features are drawn to scale. **(C)** *AtP4H3* transcript levels in wild type, T-DNA mutant line (*p4h3-2*) and complementation (compl) line #2 in 7 day-old seedlings. The RT-PCR was performed with qPCR primers. Actin was used as internal control. **(D)** Relative expression of *AtP4H3* in Col-0 (green bars) and *p4h3-2* mutant (gold bars) plants in response to a hypoxic time course by using qPCR. Relative expression levels are shown as fold change values and the error bars represent standard errors. **(E)** Phenotypes of the wild type (Col-0), *p4h3-2*, and complementation line 2 exposed to 100% N_2_ for 7.5 h after a 3-day post-anoxic recovery. **(F)** Survival rates of 7 day-old Arabidopsis plants after 100% N_2_ treatment for 7.5 h. Fifty seeds were placed per 10 mm Petri dishes. Survival rates were scored as a percentage following 3 days of post-anoxic recovery under normal conditions (21% O_2_, air). Data are shown as mean survival rates, error bars show Standard error. The experiment was repeated at least three times.

The tissue-specific expression of *AtP4H3* was determined in various organs and developmental stages by RT-PCR (Figure 1A). The *AtP4H3* was expressed at the seedling stages (1–3 weeks) in both roots and leaves, in young inflorescences (6 weeks) and siliques (8 weeks), while there was no expression at the seed stage and only minimal at 8 week-old inflorescences (Figure 1A).

The physiological significance of AtP4H3 under oxygen deficiency conditions was investigated by characterizing the anoxic response of a *p4h3-2* T-DNA line (Figure 1B). This mutant line was carrying an insertion at the 4th exon of *AtP4H3* (SALK_076446) which was verified by genotyping (Figure 1B). The transcript abundance of *AtP4H3* was determined in 7-day old seedlings of wild type, *p4h3-2* T-DNA line and *AtP4H3* complementation line #2 (*MAITG3-2B*) by using a RT-PCR approach (Figure 1C). No expression was observed in the *p4h3-2* line while mRNA levels were detected in wild type and *MAITG3-2B* (Figure 1C). Homozygous *p4h3-2* plants developed aerial lateral inflorescences inconsistently and further investigation will be required to characterize the phenotype (Supplementary Figure 1).

Seven day-old seedlings of wild type Col-0 and *p4h3-2* were exposed to hypoxia and the *AtP4H3* transcript levels were determined (Figure 1D). Low oxygen induced the *AtP4H3* expression in wild type throughout the 6-h time course, as expected, peaking after 1 h of hypoxia, while no detectable mRNA levels were observed for the *p4h3-2* line (Figure 1D).

Hypoxia tolerance assays indicated that Arabidopsis can easily survive at 1% O_2_ in the dark for several days (Licausi et al., 2010). The 7 day-old seedlings of *p4h3-2* line, *MAITG3-2B* and wild type were exposed to anoxia for 7.5 h in order to determine their survival percentage (Figures 1E,F). After a 3-day post-anoxia period, most of the p4h3-2 seedlings turned white and died while most of the Col-0 and *MAITG3-2B* seedlings were green and continued to grow (Figures 1E,F). The *p4h3-2* survival percentage was very low at the levels of 10.66% while in Col-0 and *MAITG3-2B* it was much higher at the levels of 44.74% and 46.46%, respectively (Figures 1E,F).

### Tissue Specific Localization of *AtP4H3* Under Hypoxia

**FIGURE 2:**
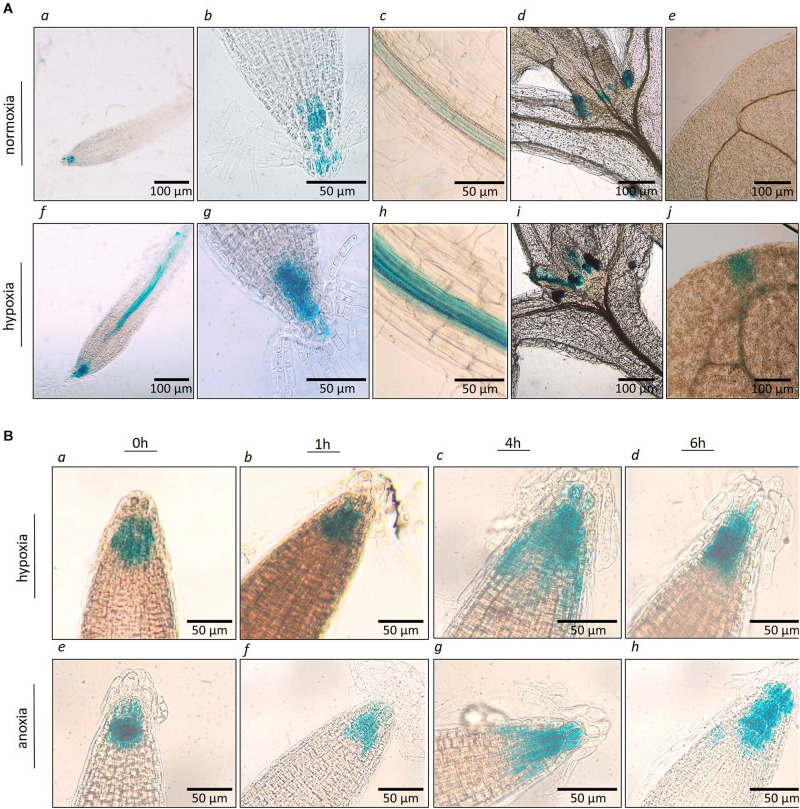
**(A)** Histochemical localization of *AtP4H3* promoter-GUS expression in 5 day-old transgenic Arabidopsis seedlings treated with 21% **(a–e)** and 1.5% O_2_ *(f–j)* for 3 h. **(a,f)** *AtP4H3* promoter activity in columella cells and vasculature in differentiation and elongation root zones. **(b,g)** *AtP4H3* promoter activity in columella cells in root tips. **(c,h)** *AtP4H3* promoter activity in the stele. **(d,i)** *AtP4H3* promoter activity in shoot apex region. **(e,j)** *AtP4H3* promoter activity in leaf tips. The assay consisted of three biological replicates, with each biological replicate including 3–5 technical replicates. **(B)** Histochemical localization of *AtP4H3* promoter-GUS expression in 7 day-old transgenic Arabidopsis seedlings treated with hypoxia (1.5% O_2_) and anoxia. **(a–d)** *AtP4H3* promoter activity in root tips under hypoxia (1.5% O_2_) time-course. **(e–h)** *AtP4H3* promoter activity in root tips under anoxia time-course.

The spatial and temporal expression of *AtP4H3* in young seedlings subjected to hypoxia and anoxia was investigated by analyzing stable Arabidopsis transgenic lines transformed with an *P4H3pro:GUS:GFP* construct. Several independent transgenic lines were generated and three of them were selected for further characterization. GUS staining indicated tissue specific expression of *AtP4H3* consistently in the root stele (Figures 2A,c,f,h), root tips (Figures 2A,g), stipules (Figures 2A,d,i), and leaf tips (Figures 2A,e,j) of 5-day old seedlings. Hypoxia treatment for 3 h significantly increased GUS expression in columella cells, root stele and stipules while strong induction was observed in the stele of the root tip differentiation zone (Figure 2A). Moreover, a 1.5% O_2_ hypoxic and an anoxic time course experiment was performed to determine GUS expression patterns in the root tip (Figure 2B). The highest upregulation was observed after 4 h of hypoxia and anoxia with detection of GUS staining also in lateral root cup in addition to columella cells (Figure 2B). These results indicate that *AtP4H3* is induced under oxygen deficiency conditions within 4 h of exposure and this induction is sustained up to 6 h (Figure 2).

### *AtP4H3* Alters the Expression of Anaerobic Marker Genes

**FIGURE 3:**
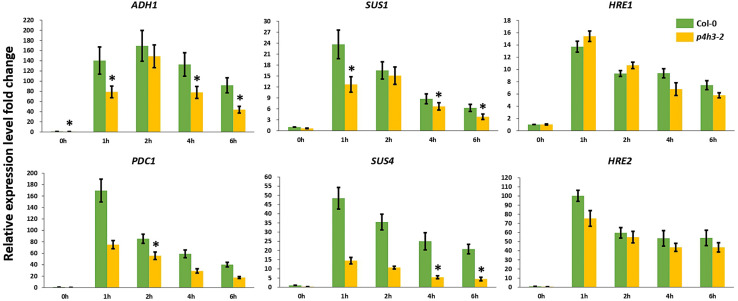
Expression of anaerobic marker genes under hypoxia. The expression of six genes (*ADH1*, *PDC1*, *SUS1*, *SUS4*, *HRE1*, and *HRE2*) was analyzed under hypoxia (1.5% O_2_) in 7 days-old Arabidopsis seedlings: Col-0 (green bars) and *p4h3-2* (gold bars). Relative expression levels are shown as fold change values. Asterisks represents statistical significance between Col-0 and *p4h3-2* within a treatment and the error bars represent the standard error in expression values.

Considering the significantly lower survival percentage of *p4h3-2* line, the possible effect of *AtP4H3* on anaerobic response marker genes was investigated, despite the fact that AtP4H3 catalyzes a post-translational modification. The *ADH1*, *PDC1*, *SUS1*, *SUS4* and the TFs, *HRE1*, and *HRE2*, were selected for expression analysis during a 1.5% O_2_ hypoxic time course by using qPCR analysis (Figure 3). The transcript levels of *ADH1*, *PDC1*, *SUS1*, and *SUS4* were down-regulated in the mutant compared to Col-0 control for most of the 1-, 2-, 4-, and 6-h time points (Figure 3). The *ADH1* expression, for most time points, was decreased by 50–60 fold in the *p4h3-2* line, while lower fold change induction was observed for *PDC1*, *SUS1*, and *SUS4* compared to Col-0 (Figure 3). Strong induction by 14- and 100-fold was detected in *HRE1* and *HRE2* transcript abundance in response to hypoxia, respectively (Figure 3). However, similar *HRE1* and *HRE2* mRNA levels were detected in both *p4h3-2* and Col-0 seedlings, indicating that these hypoxia inducible TFs were not involved in the differential expression patterns of the anaerobic marker genes in *p4h3-2* line under 1.5% O_2_ conditions (Figure 3).

### Oxygen Deficiency Induces AtP4H3-Dependent Changes in AGP Gene and Protein Expression Profiles

Several Arabidopsis and tomato AGPs were shown to be differentially expressed under low oxygen and among them nine Arabidopsis AGPs were selected for further characterization considering microarray analysis data of low oxygen transcriptome in Arabidopsis (Liu et al., 2005; Showalter et al., 2010; Fragkostefanakis et al., 2012). This cluster of hypoxic AGPs is comprised of five classical AGPs and four (Fasciclin-like AGPs) FLAs (Supplementary Figure 2). All of them comprise signal peptides while the five classical AGPs and two FLAs, FLA8 and FLA12, contain a GPI anchor (Supplementary Figure 2).

**FIGURE 4:**
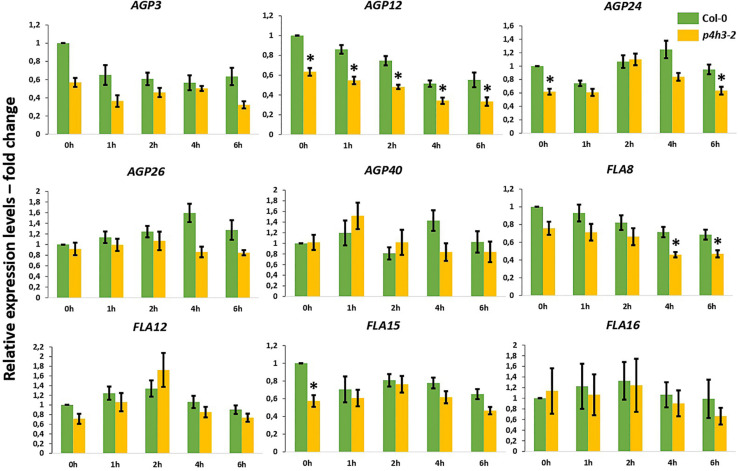
Expression of AGPs genes under hypoxia in the *p4h3-2* line. The expression of nine AGPs – *AGP3*, *AGP12*, *AGP24*, *AGP26*, *AGP40*, *FLA8*, *FLA12*, *FLA15*, and *FLA16* – was measured in hypoxic conditions (1.5% O2) in 7 day-old Arabidopsis seedlings. Col-0 (green bars) and *p4h3-2* (gold bars) relative expression levels are shown as fold change values. Asterisks represent difference of significance between Col-0 and *p4h3-2* within a treatment and error bars represent the standard error in expression values.

Stable overall transcript levels of AGPs and FLAs were observed in the wild type Col-0 seedlings throughout the 6-h hypoxic time course by qPCR analysis (Figure 4).

In the *p4h3-2* line, the *AGP12* showed lower levels of expression under normoxia (0 h) and this downregulation was continued compared to Col-0 throughout the time course (Figure 4). The *FLA8* and *AGP24* showed lower transcript levels after 4-, 6-h, and 6-h of hypoxia, respectively (Figure 4). These results might suggest indirect involvement of AtP4H3 also on the transcriptional regulation of AGPs despite catalyzing a post-translational modification. Comparison of the expression levels among the nine AGPs showed that *AGP24* and *AGP3* had the highest transcript abundance under normoxia (0 h) which was sustained also under hypoxia (Supplementary Figure 3). In the *p4h3-2* mutant background, the mRNA levels of AGP24 increased by 2-fold in one time point (Supplementary Figure 3).

**FIGURE 5:**
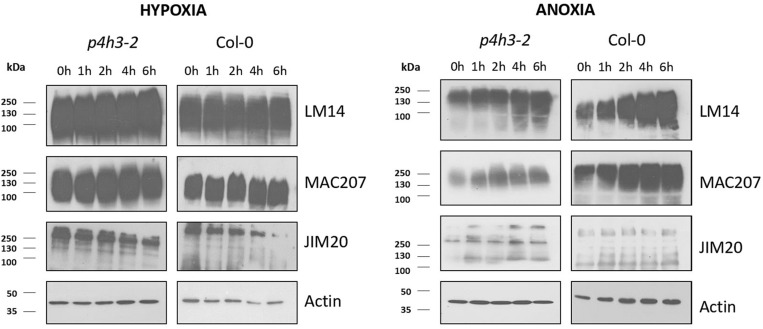
Western blot analysis of *p4h3-2* and Col-0 seedlings using the LM14, MAC207, and JIM20 antibodies of AGPs. Total proteins (10 μl per lane) from Col-0 and *p4h3-2* plants following hypoxia **(left panel)** and anoxia **(right panel)** treatments. Total proteins extracted from roots of 7 day-old seedlings subjected to hypoxia or anoxia for 0, 1, 2, 4, and 6 h were fractionated in SDS-PAGE in triplicate. Molecular masses are indicated on the left (kDa). At least three biological replicates were performed.

The soluble AGPs and extensins comprising the LM14-, MAC207-, and JIM20-epitopes were determined by western blot analysis (Figure 5). The LM14 antibody recognizes AGP-bound epitopes containing neutral sugars such as arabinose and galactose and are abundant in their side chains (Moller et al., 2008), while the MAC207 recognizes an epitope containing L-arabinose and o-glucuronic acid (Pennell et al., 1989). The JIM20 antibody recognizes extensin or HRGP glycoproteins (Knox et al., 1995).

The soluble AGPs comprising the LM14-, MAC207-, and JIM20-epitopes ranged from 250 up to 72 KDa (Figure 5). The LM14-bound AGPs showed stable protein levels in wild type Col-0 during the hypoxic time course and a marginal increase during the anoxic time course (Figure 5 and Supplementary Figure 4). Similar results were obtained in the *p4h3-2* mutant background (Figure 5 and Supplementary Figure 4). The MAC207-bound epitopes exhibited stable levels of expression under hypoxic time course in wild type Col-0 and the *p4h3-2* mutant line while a marginal upregulation was observed under anoxia (Figure 5 and Supplementary Figure 4). The JIM20-bound extensins exhibited stable levels with a slight decrease after 6 h of hypoxia in wild type Col-0 while under anoxia the JIM20 levels were stable (Figure 5 and Supplementary Figure 4). In the *p4h3-2* mutant background, the JIM20 epitopes slightly decreased under hypoxia and increased under anoxia (Figure 5 and Supplementary Figure 4).

An anoxic time course of up to 12 h in wild type Col-0 and *p4h3-2* mutant indicated slightly higher levels of LM14 and MAC207 epitopes suggesting possible contribution to cell homeostasis (Supplementary Figure 5). Marginally higher levels of AGPs-bound epitopes were detected in the wild type Col-0 compared to the *p4h3-2* mutant line under anoxic conditions in two different anoxic time courses (Figure 5 and Supplementary Figures 4, 5) while no changes in AGPs content was observed under hypoxia (Figure 5 and Supplementary Figure 4).

## Discussion

Although several P4Hs are upregulated under oxygen deficiency, the role of proline hydroxylation on hypoxic response in plants has not been thoroughly investigated despite their role in metazoan oxygen sensing mechanism (Vlad et al., 2007a). Oxygen sensing mechanisms have been discovered in animal and plant kingdoms, while hypoxia sensing and response mechanisms utilize post-translational modifications to signal protein degradation in an oxygen dependent manner (Kaelin and Ratcliffe, 2008; Gibbs et al., 2011; Weits et al., 2014). The hydroxylation of proline residues generates a binding site for the von Hippel-Lindau (pVHL) tumor suppressor protein, which is a component of the ubiquitin ligase complex and thus, HIFa is polyubiquitylated and subjected to proteasomal degradation upon oxygen availability due to dependence of P4H activity on oxygen concentration (Kaelin and Ratcliffe, 2008; Zhang and Yang, 2012). Under hypoxia, HIFa accumulates, dimerizes with an HIFβ family member, translocates to the nucleus, and transcriptionally activates an array of genes (Mcneill et al., 2002; Kaelin and Ratcliffe, 2008). Plants have evolved another oxygen sensing system, which is comprised of different components. Instead of proline hydroxylation, the signaling modification is cysteine oxidation by PCO (Weits et al., 2014), while the proteolysis (PRT)6 N-degron pathway, equivalent to the non-plant Arg/N-degron pathway, is used for proteasomal degradation (Vicente et al., 2017; Dissmeyer, 2019).

The lower survival in the *p4h3-2* line was associated with a significant downregulation trend of *ADH* and *PDC1* expression (Figure 3). It is well established that increased activation of the fermentation pathway leads to better adaptation under oxygen deficiency (Ismond et al., 2003; Hwang et al., 2011; Mithran et al., 2014). Lower survival of Arabidopsis seedlings in *hre1/hre2* mutant lines were associated with lower expression levels of *ADH* in two out of four time points in an 8-h hypoxic course (Licausi et al., 2010). In the *p4h3-2* line, the decrease in survival of Arabidopsis seedlings was also associated with a suppression of *ADH* and *PDC1* expression in three and one time point in a 6-h hypoxic time course, respectively (Figure 3). Moreover, the transcript levels of *SUS1* and *SUS4* were also downregulated in two time points in an 8-h hypoxic course in the *hre1/hre2* mutant lines (Licausi et al., 2010). Similarly, in the *p4h3-2* line, the expression of *SUS1* and *SUS4* was decreased in three and two time points, respectively (Figure 3). These results indicate similar responses to hypoxia, which might not be related to either HRE1 and/or HRE2, because their levels of expression were not altered in the *p4h3-2* line (Figure 3).

One of the anaerobic genes, *SUS1*, was shown to be expressed in the phloem and contribute to hypoxia and anoxia tolerance, indicating partially similar to *AtP4H3* spatial expression patterns (Martin et al., 1993; Yao et al., 2020). However, *sus1/sus4* double mutants had significantly lower survival percentages under waterlogging, while similar to wild type survival was observed under anoxia, hypoxia and submergence (Bieniawska et al., 2007; Santaniello et al., 2014).

The spatial induction of *AtP4H3* in root stele and columella cells, leaf tips and stipules of Arabidopsis seedlings subjected to hypoxia suggests tissue specific involvement in hypoxic adaptation. The strongest induction was observed in root stele and columella cells, while it has been reported that the respiration rates in stele of aerated roots were 6-fold higher in comparison to cortex tissue in banana plants (Aguilar et al., 2003), which indicates accelerated consumption of oxygen and therefore induction of oxygen deficiency in the stele under aerobic conditions. This suggest that the level of *AtP4H3* induction might be regulated in an oxygen dependent manner.

The HRGP superfamily in Arabidopsis consists of 166 genes including 85 genes coding for various AGP subfamily members, from which 22 genes code for classical AGPs and 21 for FLAs (Showalter et al., 2010). Hypoxia and anoxia differentially regulate AGPs expression in tomato fruits, with transcript levels of some tomato AGPs significantly decreased under hypoxia and up- or down-regulated under anoxia (Fragkostefanakis et al., 2012). Among the five classical AGPs and the four FLAs, only *AGP12* showed consistently lower expression levels in the *p4h3-2* line throughout the 6-h hypoxic time course (Figure 4), indicating regulation at the transcriptional level despite the fact that *AtP4H3* catalyzes a post-translational modification. This pattern of expression might be attributed to regulation by a transcription factor which is affected by the levels of expression of AtP4H3. In this case, the AtP4H3 might indirectly regulate the expression of AGP12. Overall, the other eight AGPs and FLAs exhibited stable levels of expression throughout the hypoxic time course Stable levels of gene expression (Figure 4) and protein content (Figure 5) were detected for the other eight AGPs and FLAs and the AGPs-bound epitopes during the hypoxic time course, respectively.

Previously, 25 TFs were identified to exhibit differentially expression patterns under hypoxia and were comprising proline hydroxylation motifs in their deduced amino acid sequence (Vlad et al., 2007b). However, there are no reports, up to now, on the hydroxylation of TFs in plants regulating protein stability. Alternatively, secreted peptide hormones such as PSY1, CEPs, CLV3, CLEs, CLE-PS2, and RGFs, which undergo posttranslational hydroxylation (Lee et al., 2020), might be involved in hypoxic response and regulate transcriptional responses through TFs. Among them, CLE and CEP peptides were shown to be associated with abiotic stress such as osmotic and drought tolerance in Arabidopsis (Smith et al., 2020; Takahashi et al., 2020).

The frequency of occurrence of proline hydroxylation determines the O-glycosylation of AGPs and extensins leading to either alterations of their structure and/or to their degradation. This might lead to marginal reduction in the content of AGP-bound epitopes in the *p4h3-2* line in comparison to the wild type as observed in the anoxic time course. However, no changes in the AGPs content was detected between the *p4h3-2* line and wild type in the hypoxic time course possibly due to redundant function of other P4Hs expressed under hypoxia.

A marginal, gradual decrease on the extensins-bound epitopes was detected throughout the hypoxic time course in both *p4h3-2* line and wild type suggesting lower involvement in hypoxic response compared to AGPs. However, under anoxia an induction was observed only in the *p4h3-2* line but not in the wild type indicating possible regulation either at the transcriptional level or alterations at their glycan structure. Further investigation of the transcriptome and proteome might provide additional information on the regulation of extensin levels under oxygen deficiency conditions.

Mainly anoxic but also hypoxic stress leads to a substantial decrease in ATP production and as a result to energy shortage (Gibbs and Greenway, 2003). In this context, the gradual increase in AGPs content throughout not only the 6-h but also the 12 h anoxic time course might suggest participation in the preservation of cell homeostasis under anoxia considering that protein synthesis is a high energy consumption cellular process (Branco-Price et al., 2005). The higher sensitivity of an *AtP4H3* knock out mutant line to anoxic stress indicates the physiological significance of proline hydroxylation, a post-translation modification, in oxygen deficiency adaptation in Arabidopsis. Further investigation is required on the identification of proline hydroxylated proteins involved in this abiotic stress response.

## Data Availability Statement

The original contributions presented in the study are included in the article/Supplementary Material, further inquiries can be directed to the corresponding author.

## Author Contributions

PK conceived and designed the work. PK, AKo, MK, AL, ZZ, AKr, MB, MI, ME, GT, PC, NP, and A-MD were involved in the experiments, analysis, and interpretation of the data. PK, AKo, MK, AL, ZZ, AKr, and MI were involved in drafting the work. PK, AKo, and MI revised and approved the final version. All authors contributed to the article and approved the submitted version.

## Conflict of Interest

The authors declare that the research was conducted in the absence of any commercial or financial relationships that could be construed as a potential conflict of interest.

## Footnotes

1 http://arabidopsis.info

2 http://www.ncbi.nlm.nih.gov/projects/gorf

3 http://www.invitrogen.com

4 http://www.psb.ugent.be/gateway
