# Supplementary material for: An Arabidopsis Prolyl 4 Hydroxylase Is Involved in the Low Oxygen Response
Source: Front Plant Sci. 2021 Mar 15;12:637352. doi: 10.3389/fpls.2021.637352 (PMC8006943; doi:10.3389/fpls.2021.637352)
Supplement: Supplementary file 1 [file Data_Sheet_1.pdf]

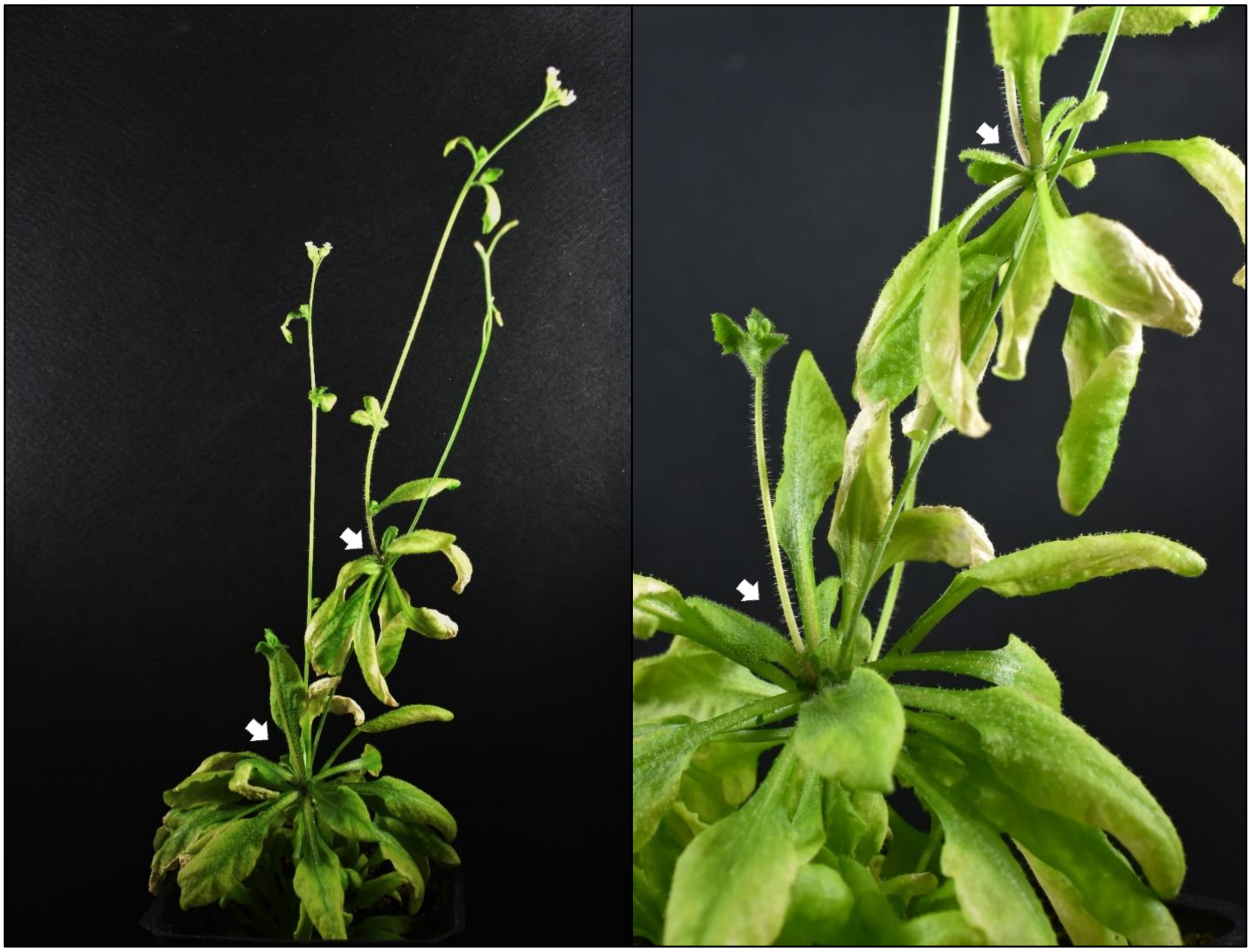

Figure S1. Loss of *AtP4H3* function likely results in the formation of lateral aerial rosettes. Eight week old *p4h3-2* plants with lateral aerial rosettes indicated by white arrows.

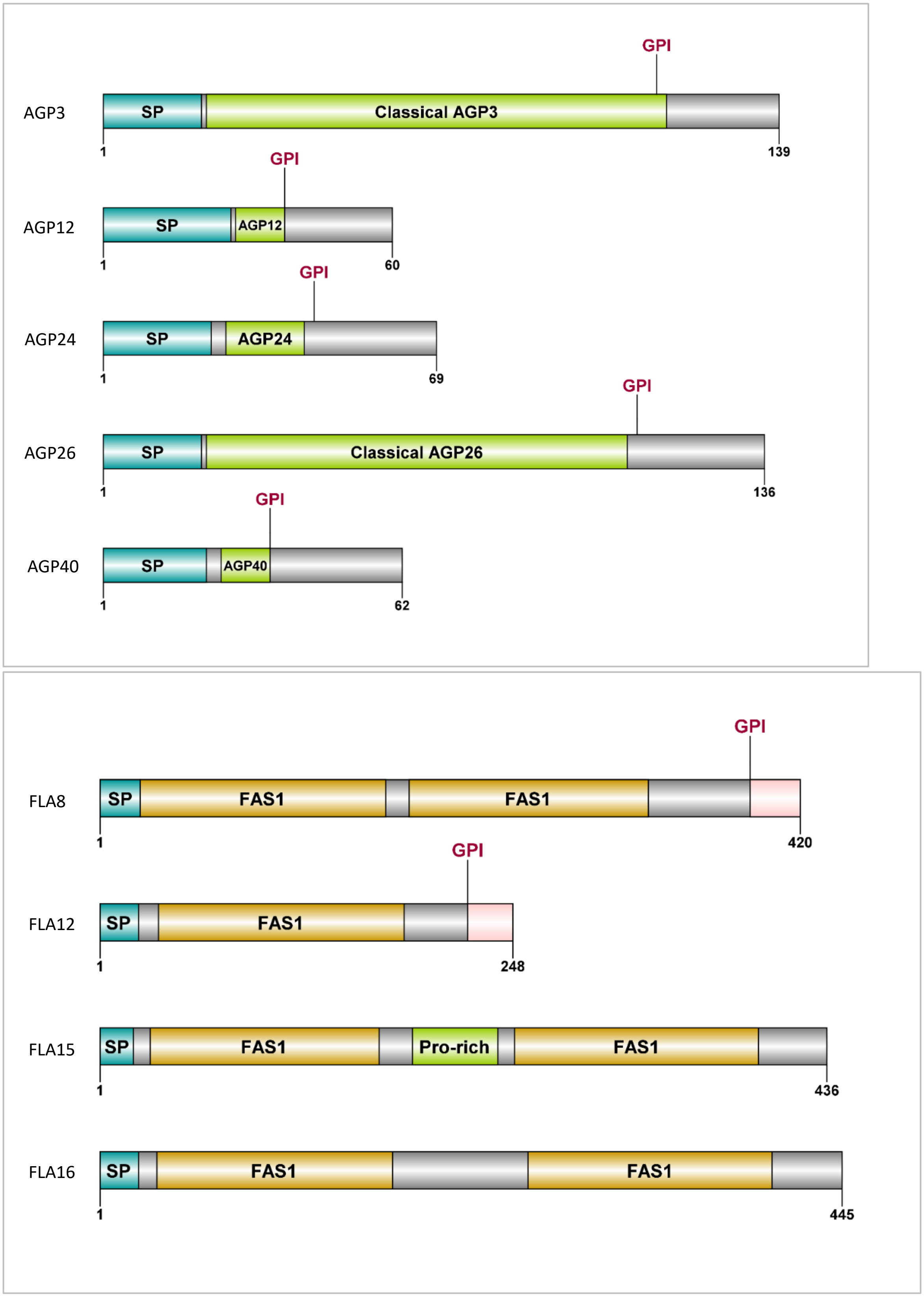

Figure S2. AGP and FLA protein structures. (Top Panel) AGP3, AGP12, AGP24, AGP26 and AGP40 protein structure. All AGPs have an N-terminal signal peptide (SP), targeting the protein to ER and eventually determining its secretion fate or location along the secretory pathway, and GPI anchor.

Hydroxylated Proline residues: Pro-34, Pro-36, Pro-38 and Pro-40 in AGP24(28), Pro-32, Pro-34 and Pro-36 in AGP12(28,29), Pro-28, Pro-30 and Pro-32 in AGP40(28). (Bottom Panel) FLA12, FLA8, FLA16, and FLA15 protein structures. All the FLAs have an N-terminal signal peptide (SP), targeting the protein to ER and eventually determining its secretion fate or location along the secretory pathway and FAS1 (Fasciclin-like arabinogalactan protein) domains. FLA12 and FLA8 sequences include GPI anchor. All protein structures were designed according to https://www.uniprot.org/ using DOG 1.0 software (http://bioinformatics.lcd-ustc.org/dog).

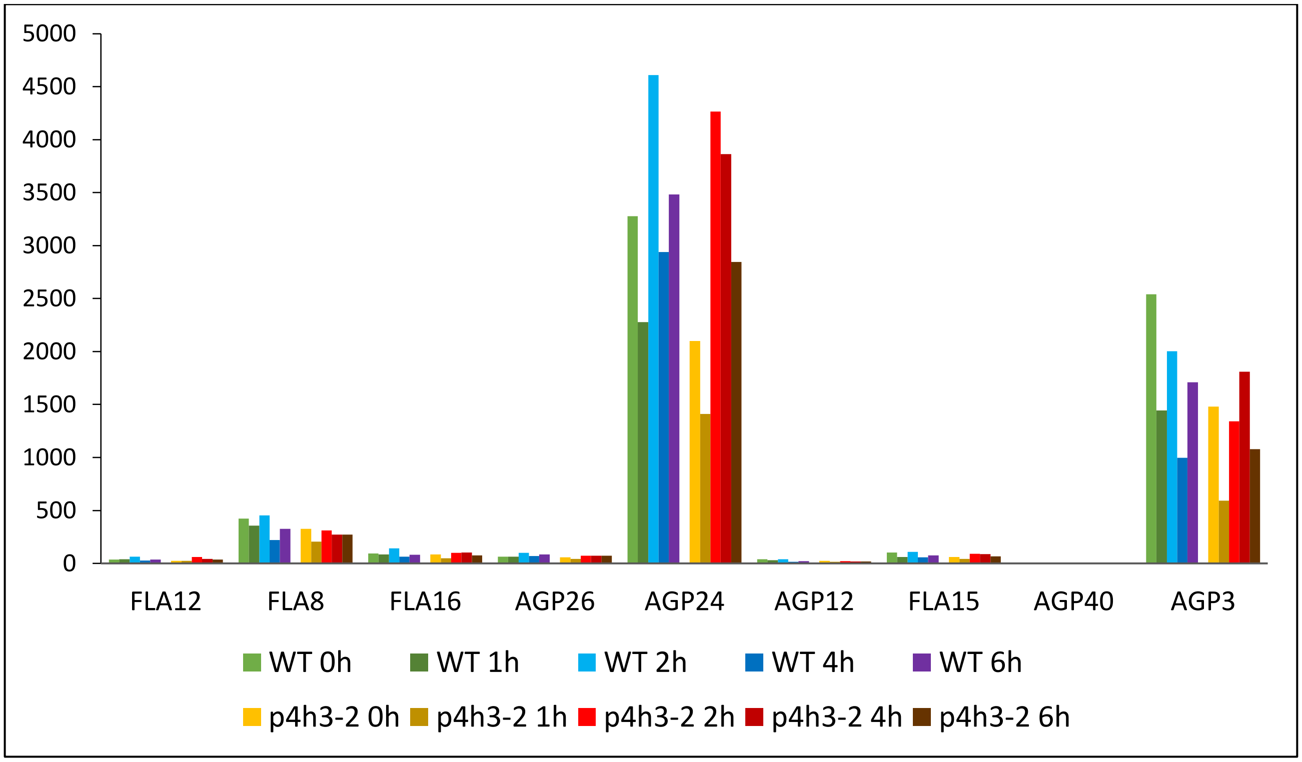

Figure S3. Relative expression of AGPs under hypoxia. The expression of nine Arabidopsis AGPs (*FLA12*, *FLA8*, *FLA16*, *AGP26*, *AGP24*, *AGP12*, *FLA15*, *AGP40* and *AGP3*) was measured under hypoxia (1.5% O_2) in root tissue from 7 day-old Arabidopsis seedlings by qPCR. Col-0 (WT) and *p4h3-2* relative expression levels are shown as fold change values; *AGP40* was used as control for each time-point of both Col-0 and *p4h3-2*.

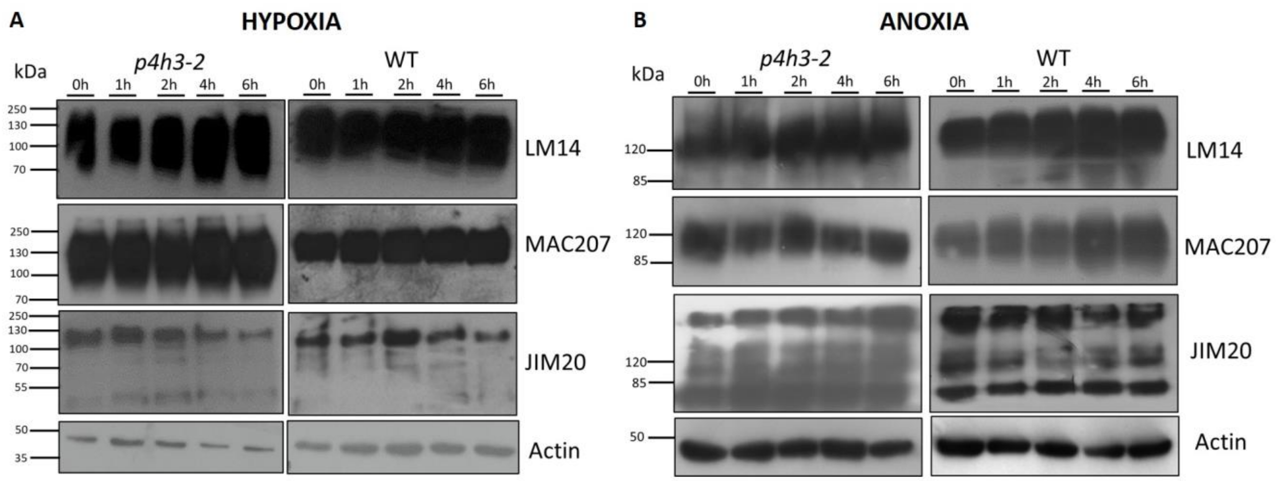

Figure S4. Western blot analysis of *p4h3-2* and Col-0 seedlings under hypoxia and anoxia by using the LM14, MAC207, and JIM20 antibodies. Total proteins were extracted and subjected to hypoxia (A) or anoxia (B) for 0, 1, 2, 4 and 6 hours and fractionated in SDS-PAGE of 12% and 8%, respectively. Molecular markers are indicated on the left (kDa).

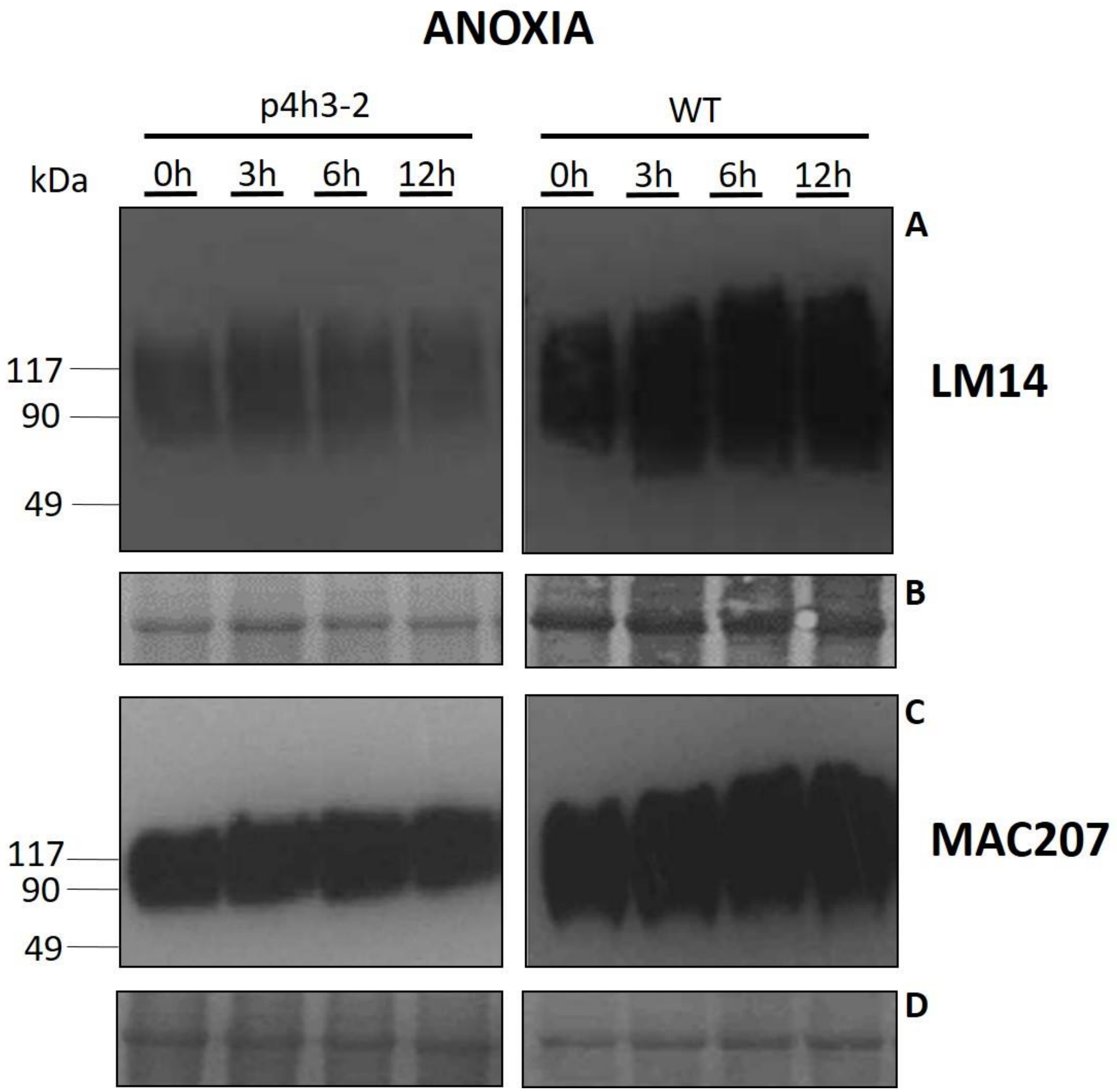

Figure S5. Western blot analysis of *p4h3-2* and Col-0 seedlings under anoxia by using the LM14 (A) and MAC207 (C) antibodies. Total proteins were extracted and subjected to anoxia for 0, 1, 3, 6 and 12 hours and fractionated in 12% SDS-PAGE. (A) LM14- and (C) MAC207-bound eitopes, (B) and (D) Coomassie Blue-stained polyacrylamide gel. Molecular markers are indicated on the left (kDa).
