# Supplementary material for: An Arabidopsis Prolyl 4 Hydroxylase Is Involved in the Low Oxygen Response
Source: Front Plant Sci. 2021 Mar 15;12:637352. doi: 10.3389/fpls.2021.637352 (PMC8006943; doi:10.3389/fpls.2021.637352)
Supplement: Supplementary file 2 [file Table_1.DOCX]

**Table S1. Primer sequences used in construction of transgenic lines and expression analysis**

| Primer | Sequence |
| --- | --- |
| PromP4H3attB2 | GGGGACCACTTTGTACAAGAAAGCTGGGTTCAACAAAGATCCACCCGAGATG |
| PromP4H3attB1 | GGGGACAAGTTTGTACAAAAAAGCAGGCTCATGATAGTGATGATAAATCGCAGA |
| attB1-Prom-P4H3 | GGGGACAAGTTTGTACAAAAAGCAGGCTACAAAACAAAAACAGGGAAAC |
| attB2-UTR3-P4H3 | GGGGACCACTTTGTACAAGAAAGCTGGGTAATAATAATCTCTTCCAACAC |
| EGFP | GAACTTCAGGGTCAGCTTGC |
| GUSus | CCTTTCTTGTTACCGCCAACG |
| M13-Forward | GTAAAACGACGGCCAG |
| M13-Reverse | CAGGAAACAGCTATGAC |
| P4H-D3F | TTTCAAGCGAGGAAATGGTC |
| Co-P4H3-LP | ATTTTGTACTCGCCAACATGC |
| LBb1 | GCGTGGACCGCTTGCTGCAACT |
| AtP4H3-rt-F | GCTTTGATGGGATTCAAGGTTTATC |
| AtP4H3-rt-R | GTCACTCTAAGCCATCCTAATTTACTC |
| Adh1-RT-F | GAAATTCATCACTCACACAGTGCCAT |
| Adh1-RT-R | TCAAGCACCCATGGTGATGATG |
| AtPDC1-rt-F | GCGCTTCTTACCAATTTAGTGACAT |
| AtPDC1-rt-R | GCCCATGATAAAGCGTACATGGAA |
| AtSUS-1-F | GACTCGGAACCAGTAAAGAGTTT |
| AtSUS-1-R | GCGCTTCCCAATCCGAATGATA |
| AtSUS-4-F | GCCGAAGATTTTCTACATTCTACAAC |
| AtSUS-4-R | GCCAAACAATGGTGAAGCATATCAT |
| AtHRE1-RT-F | CCAAGGCTAAGACTGTGCAAC |
| AtHRE1-RT-R | CCGGATTATTCTCCTCCCACA |
| AtHRE2-RT-F | AGAAGAAGAAGCCGATACTAAACCA |
| AtHRE2-RT-R | TCATGTAATCCTCCAATGCCATCA |
| FLA12_F_rt | ACTCCGCCGATGGACATTTT |
| FLA12_R_rt | TGTAGACATTGCCGGAGACG |
| FLA8_F_rt | TCAGACTCGCCAACAGGTTC |
| FLA8_R_rt | GAGACACGGAGACGGCTATG |
| FLA16_F_rt | CCAACGACCATCTCCACCTC |
| FLA16_R_rt | GACGTTCGATGCCGTGAATG |
| AGP26_F_rt | TGAGTTTCAGCTGTCCACCA |
| AGP26_R_rt | GTAGGGAACAGGGGAGAGGT |
| AGP24_F_rt | TGGTTTGTCTATTGGCGACGA |
| AGP24_R_rt | AACGGTTGAGCTAGATGCGG |
| AGP12-FW_rt | GCCACAACTCATCATTCGCA |
| AGP12-R_rt | GCGGAGAAAGCCACAATAGC |
| FLA15-F_rt | CAATGTCACCTGGTCCGTCA |
| FLA15-R_rt | AGCTTCGTCGTTTGGTGCTA |
| AGP40-F_rt | GTCTCCGCGGCTACAATGG |
| AGP40-R_rt | CCACCGGAAAAGCTACGGT |
| AGP3-F_rt | TCCGGCACCTATCACGTTTC |
| AGP3-R_rt | GAGACGGTTGGTGGAGTTGT |
